# Chromogranin A cell density in the rectum of patients with irritable bowel syndrome

**DOI:** 10.3892/mmr.2012.1087

**Published:** 2012-09-18

**Authors:** M. EL-SALHY, T. MAZZAWI, D. GUNDERSEN, T. HAUSKEN

**Affiliations:** 1 Section for Gastroenterology, Department of Medicine, Stord Helse-Fonna Hospital, Stord; 2 Section for Gastroenterology, Institute of Medicine, University of Bergen, Bergen; 3 Department of Research, Helse-Fonna, Haugesund, Norway

**Keywords:** chromogranin A, computer image analysis, immunohistochemistry, irritable bowel syndrome, rectum

## Abstract

In a previous study, chromogranin A (CgA) cell density in the colon of patients with irritable bowel syndrome (IBS) was found to be reduced. It has been suggested that intestinal CgA cell density may be used as a marker for the diagnosis of IBS. The rectum harbours a larger number of large intestinal endocrine cells and is more accessible for biopsies than the colon. The present study aimed at determining the CgA cell density in the rectum of IBS patients. A total of 47 patients with IBS that fulfilled the Rome Criteria III (39 females and 8 males; average age, 38 years) were included. A total of 28 patients had diarrhea (IBS-D) and 19 had constipation (IBS-C) as the predominant symptom. A total of 27 subjects that underwent colonoscopy with rectal biopsies were used as the controls. These subjects underwent colonoscopy due to gastrointestinal bleeding (the source of which was identified as haemorrhoids or angiodysplasia; 19 females and 8 males; average age, 49 years), or health worries. The rectal biopsies were immunostained for CgA and quantified by computer image analysis. The CgA density in the controls was 206.3±22.2 (mean ± SEM), in all IBS patients 190.2±14.3, in IBS-D patients 188.8±14.7 and in IBS-C patients 195.3±34.1. There was no statistically significant difference between the controls, IBS, IBS-D or IBS-C patients (P=0.5, 0.5 and 0.7, respectively). The present study showed that although the rectum comprises the same endocrine cell types as the colon, attention must be paid when drawing conclusions regarding the whole large intestine from studies carried out on the rectum. This particularly applies when endocrine cells are investigated. As CgA cell density represents the total endocrine cell content of the rectum, changes in specific endocrine cells in IBS patients cannot be excluded.

## Introduction

Irritable bowel syndrome (IBS) is a chronic gastrointestinal disorder affecting 5–20% of the world’s population. IBS symptoms include abdominal discomfort or pain associated with altered bowel habits and often bloating and abdominal distension (1). IBS is not known to be associated with the development of serious diseases or with excess mortality (2,3). However, it considerably reduces the quality of life (1). Apart from the increased morbidity caused by IBS, it is an economic burden to society in a number of ways (a rise in sick leave or over-utilization of healthcare resources) (1).

Chromogranin A (CgA) is a marker for gut endocrine cells and endocrine tumours (4–6). It is a 68 kDa protein comprising 439 amino acid residues and was isolated for the first time from secretory granules of the bovine adrenal medulla (4,6). CgA is co-stored and co-released with monoamines and peptide hormones of the adrenal medulla, pituitary gland, parathyroid, thyroid C-cells, pancreatic islets, endocrine cells of the gastrointestinal tract and sympathetic nerves (4,5).

CgA cell density has been found to be reduced in the colon of IBS patients and it has been suggested that intestinal CgA cell density may be used as a marker for the diagnosis of IBS (1,7). The human rectum has the same endocrine cell types as the colon, namely: serotonin-, peptide YY (PYY)-, pancreatic polypeptide (PP)-, enteroglucagon- and somatostatin-producing cells (8,9). The rectum harbours, however, a larger number of these endocrine cells (8,9). In addition, the rectum is more accessible for biopsies than the colon. Therefore, the present study aimed to determine CgA cell density in the rectum of IBS patients and to examine whether biopsies would be easier and would have higher specificity and sensitivity when performed in the rectum compared to the colon.

## Materials and methods

### Patients and controls

A total of 47 patients with IBS that fulfilled the Rome Criteria III (39 females and 8 males; average age, 38 years; range, 18–65 years) were included in this study (10; http://www.romecriteria.org). A total of 28 patients had diarrhea (IBS-D) and 19 had constipation (IBS-C) as the predominant symptom. All patients underwent a complete physical examination comprising of blood tests (including full blood count, electrolytes, calcium and inflammatory markers), liver tests and thyroid function tests.

A total of 27 subjects (19 females and 8 males; average age, 49 years; range, 18–67 years), who underwent colonoscopy with rectal biopsies were used as the controls. In 20 of these subjects, colonoscopy was performed due to gastrointestinal bleeding, the source of which was identified as haemorrhoids (18 subjects) or angiodysplasia (2 subjects). The other 7 subjects were examined due to worries resulting from having relatives diagnosed with colon carcinoma.

The study was performed in accordance with the Declaration of Helsinki and was approved by the local Committee for Medical Research Ethics. All subjects provided oral and written informed consent.

### Colonoscopy

The patients, as well as the controls underwent colonoscopy, while biopsies were obtained from the rectum, approximately 15 cm from the anus. Biopsies were fixed in 4% buffered paraformaldehyde overnight, embedded in paraffin and cut into 5 μm-thick sections.

### Histopathology and immunohistochemistry

The sections were stained with haematoxylin and eosin and immunostained with the avidin-biotin complex (ABC) method using a Vectastain ABC kit (Vector Laboratories, Burlingame, CA, USA), as previously described in detail (11). The primary antibody used was monoclonal mouse anti-N-terminal of purified CgA (DakoCytomation, Glostrup, Denmark; code no. M869). The second layer biotinylated mouse anti-IgG was obtained from DakoCytomation.

### Computerized image analysis

Computerized image analysis was performed using Olympus software: Cell ^D. When using ×20 objectives, the frame (field) on the monitor represents an area of 0.14 mm^2^ of the tissue. The number of CgA immunoreactive cells and the area of the epithelial cells were measured in each field. Measurements were taken in 10 randomly chosen fields for each individual. Data from the fields were tabulated, the number of cells/mm^2^ of the epithelium was computed and statistically analysed automatically.

### Statistical analysis

Mann-Whitney U test was performed. P-values <0.05 were considered to indicate statistically significant differences.

## Results

### Colonoscopy, histopathology and immunohistochemistry

**Figure 1:**
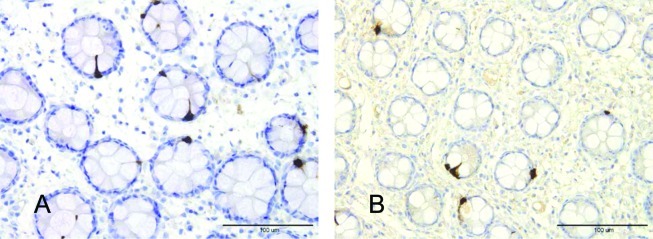
CgA-immunoreactive cells in the rectum of (A) a control and (B) a patient with IBS.

The colons of the patients and the controls were macroscopically normal. Histopathological examination of the colon biopsies from patients and controls revealed normal histology. CgA cells were located mostly in the upper part of the crypts of Lieberkühn (Fig. 1). These cells were basket- or flask-shaped.

### Computerized image analysis

**Figure 2:**
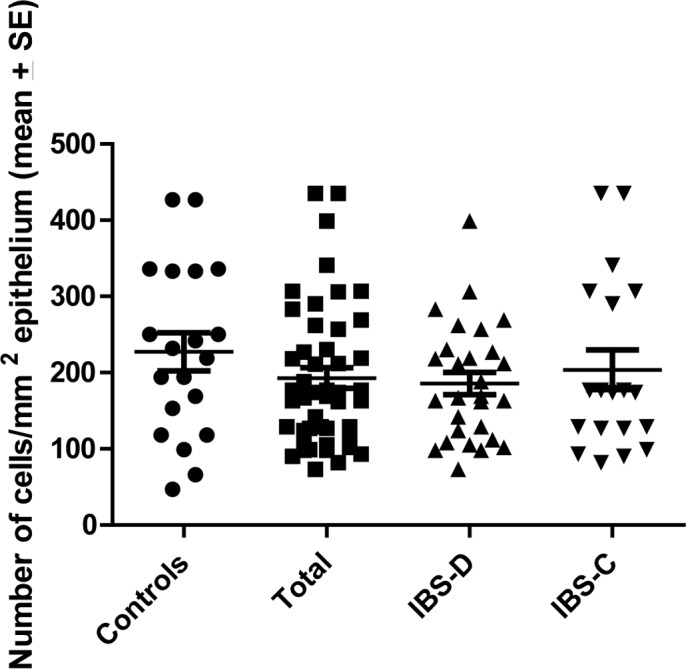
CgA-immunoreactive cell density in controls and patients with IBS.

The CgA density in the controls was 206.3±22.2 (mean ± SEM), in all IBS patients 190.2±14.3, in IBS-D patients 188.8±14.7 and in IBS-C patients 195.3±34.1 (Fig. 2). There was no statistically significant difference between the controls, IBS, IBS-D or IBS-C patients (P=0.5, 0.5 and 0.7, respectively).

## Discussion

The age and gender of the patients and healthy controls used in this study did not match completely. The control subjects were slightly older and the proportion of males to females was higher. In previous studies, however, age and gender have been found to have no effect on the density of intestinal endocrine cells in adults (8,12). The present findings demonstrating that rectal CgA cell density in patients with IBS does not differ from that of the controls was unexpected. However, taking into consideration that the colon and the rectum have different physiological functions, this observation becomes less surprising. Thus, the main function of the colon is absorbing water, sodium and some fat soluble vitamins, whereas the rectum acts as a reservoir for faeces and plays a principal role in defection (13). As mentioned previously, the rectum, besides containing the same endocrine cells as the colon, harbours a larger number of these cells as well as a larger supply of autonomic innervation compared to the colon (13).

The rectum is accessible for biopsies subsequent to a simple rigid rectoscope without any rigorous intestinal cleansing. On the other hand, biopsies from the colon require intestinal preparation, availability of a flexible colonoscope and a specialist with the knowledge of performing a colonoscopy. That is why the rectum has been chosen as a representative of the large intestine in several studies investigating endocrine cells in IBS patients (14–17). The present findings raise a warning against using the rectum as a representative of the large intestine, particularly in investigations concerning endocrine cells in a pathological condition.

Although CgA cell density, representing the total endocrine cell content of the rectum, remained unchanged in IBS patients, changes in specific endocrine cells should not be excluded. The findings asserting that changes in the serotonin cells do occur in the rectum of patients with IBS support this assumption (14–17). However, additional studies are required to clarify this hypothesis.

In conclusion, the present study demonstrates that although the rectum comprises the same endocrine cell types as the colon, attention should be paid when drawing conclusions regarding the whole large intestine from studies conducted on the rectum. This particularly applies when investigating endocrine cells.
